# Maresin1 Protect Against Ferroptosis-Induced Liver Injury Through ROS Inhibition and Nrf2/HO-1/GPX4 Activation

**DOI:** 10.3389/fphar.2022.865689

**Published:** 2022-04-04

**Authors:** Wenchang Yang, Yaxin Wang, Chenggang Zhang, Yongzhou Huang, Jiaxian Yu, Liang Shi, Peng Zhang, Yuping Yin, Ruidong Li, Kaixiong Tao

**Affiliations:** ^1^ Department of Gastrointestinal Surgery, Union Hospital, Tongji Medical College, Huazhong University of Science and Technology, Wuhan, China; ^2^ Department of Critical Care Medicine, Union Hospital, Tongji Medical College, Huazhong University of Science and Technology, Wuhan, China

**Keywords:** Maresin1, ferroptosis, reactive oxygen species, Nrf2, glutathione peroxidase 4

## Abstract

Drugs, viruses, and chemical poisons stimulating live in a short period of time can cause acute liver injury (ALI). ALI can further develop into serious liver diseases such as cirrhosis and liver cancer. Therefore, how to effectively prevent and treat ALI has become the focus of research. Numerous studies have reported Maresin1 (MaR1) has anti-inflammatory effect and protective functions on organs. In the present study, we used d-galactosamine/lipopolysaccharide (D-GalN/LPS) to establish an ALI model, explored the mechanism of liver cells death caused by D-GalN/LPS, and determined the effect of MaR1 on D-GalN/LPS-induced ALI. *In vivo* experiments, we found that MaR1 and ferrostatin-1 significantly alleviated D-GalN/LPS-induced ALI, reduced serum alanine transaminase and aspartate transaminase levels, and improved the survival rate of mice. Meanwhile, MaR1 inhibited hepatocyte death, inhibited tissue reactive oxygen species (ROS) expression, reduced malondialdehyde (MDA), reduced glutathione (GSH), GSH/oxidized glutathione (GSSG), and iron content induced by D-GalN/LPS in mice. In addition, MaR1 inhibited ferroptosis-induced liver injury through inhibiting the release of interleukin-1β (IL-1β), tumor necrosis factor-α (TNF-α), and IL-6. Subsequently, western blot showed that MaR1 improved the expression of nuclear factor E2-related factor 2(Nrf2)/heme oxygenase-1 (HO-1)/glutathione peroxidase 4 (GPX4). *In vitro* experiments, we found that MaR1 inhibited LPS-induced and erastin-induced cell viability reduction. Meanwhile, we found that MaR1 increased the MDA and GSH levels in cells. Western blot showed that MaR1 increased the expression level of Nrf2/HO-1/GPX4. Next, the *Nrf2* was knocked down in HepG2 cells, and the results showed that the protective effect of MaR1 significantly decreased. Finally, flow cytometry revealed that MaR1 inhibited ROS production and apoptosis. Overall, our study showed MaR1 inhibited ferroptosis-induced liver injury by inhibiting ROS production and Nrf2/HO-1/GPX4 activation.

## Introduction

The liver is the largest substantial organ and digestive gland in the body (Stocks et al., 2022; Yuan et al., 2022). It is closely related to the metabolism, transformation, and detoxification of the three major organic substances (Lan et al., 2022; Wang et al., 2022). Therefore, the liver occupies an important position in the body’s material metabolism. However, the incidence of acute liver injury (ALI) has gradually increased due to viral infections, improper medications, excessive intake and exposure of food additives, ethanol, ingestion of toxic foods, and radiation injury (Bechmann et al., 2012; Chao et al., 2018; LoBianco et al., 2020; Xu et al., 2020). ALI refers to acute injury or necrosis of liver cells, abnormal liver function, and even liver failure in some patients (Yang et al., 2022). Although liver cells have a strong ability to regenerate, ALI still threaten life if they are not treated in time. A better understanding of the mechanism of liver cell death is a beneficial strategy for the treatment of ALI.

Ferroptosis, an iron-dependent, novel programmed mode of cell death, was different from necrosis, apoptosis, and autophagy, and was first proposed by Stockwell in 2012 (Dixon et al., 2012; Jiang et al., 2021). The main mechanism of ferroptosis is to catalyze lipid peroxidation of unsaturated fatty acids and reduce the level of glutathione peroxidase 4 (GPX4), which induce cell death (Friedmann Angeli et al., 2014; Bersuker et al., 2019). The occurrence of ferroptosis is accompanied by the disordered iron flow and the significant increase of reactive oxygen species (ROS) (Hirschhorn and Stockwell, 2019; Su et al., 2019). Studies have reported ferroptosis is closely related to the pathological process of various diseases including tumor, neurological diseases, ischemia and reperfusion injury, hematological diseases, and kidney injury (Li et al., 2020a; Badgley et al., 2020; Ren et al., 2020; Zhong et al., 2020; Wang et al., 2021). The liver is the primary site of the storage of iron in the body, and liver damage closely linked to iron overload. Uncontrolled free iron exerts a toxic effect on the liver which can cause hepatic diseases. The use of small-molecule compounds to regulate ferroptosis, and then intervening in the occurrence of related diseases, have become a hot spot and focus of etiological research and treatment (Ding et al., 2021).

A model of ALI caused by D-galactosamine (D-GalN) and lipopolysaccharide (LPS) is widely used to study the drug development of ALI (Yang et al., 2018; Pang et al., 2020). LPS induces high secretion of pro-inflammatory cytokines and ROS production, and D-GalN is a selective hepatotoxin that disrupts hepatocyte RNA metabolism, ultimately causing liver injury. It has been reported that the ferroptosis caused by D-GalN/LPS is associated with organ injury. D-GalN/LPS-induced liver injury was accompanied with iron accumulation and ROS production, substances that regulate ferroptosis -related signaling in hepatocytes are expected to contribute to the treatment of D-GalN/LPS-induced ALI (Liu et al., 2021; Siregar et al., 2021).

Maresin1(MaR1), derived from n-3 unsaturated fatty acids, is one of the latest families of anti-inflammatory mediators. Studies have confirmed that MaR1 played a protective role in a variety of chronic inflammatory diseases by enhancing macrophage phagocytosis, reducing the production of pro-inflammatory factors and ROS, and inhibiting the nuclear factor-kappa B (NF-κB) activation (Zhang et al., 2017; Wang et al., 2020a; Zheng et al., 2020). ROS is a key mediator in the induction of ferroptosis, therefore MaR1 may inhibit ferroptosis by inhibiting ROS production caused by GalN/LPS. Furthermore, whether MaR1 alleviate D-GalN/LPS-induced liver injury through inhibiting ferroptosis has not been reported. In the present study, we investigated the roles of MaR1 in D-GalN/LPS-induced liver injury model. We demonstrated that MaR1 protected against D-GalN/LPS-induced ALI in mice by attenuating ferroptosis.

## Methods

### Animal Model

**FIGURE 1:**
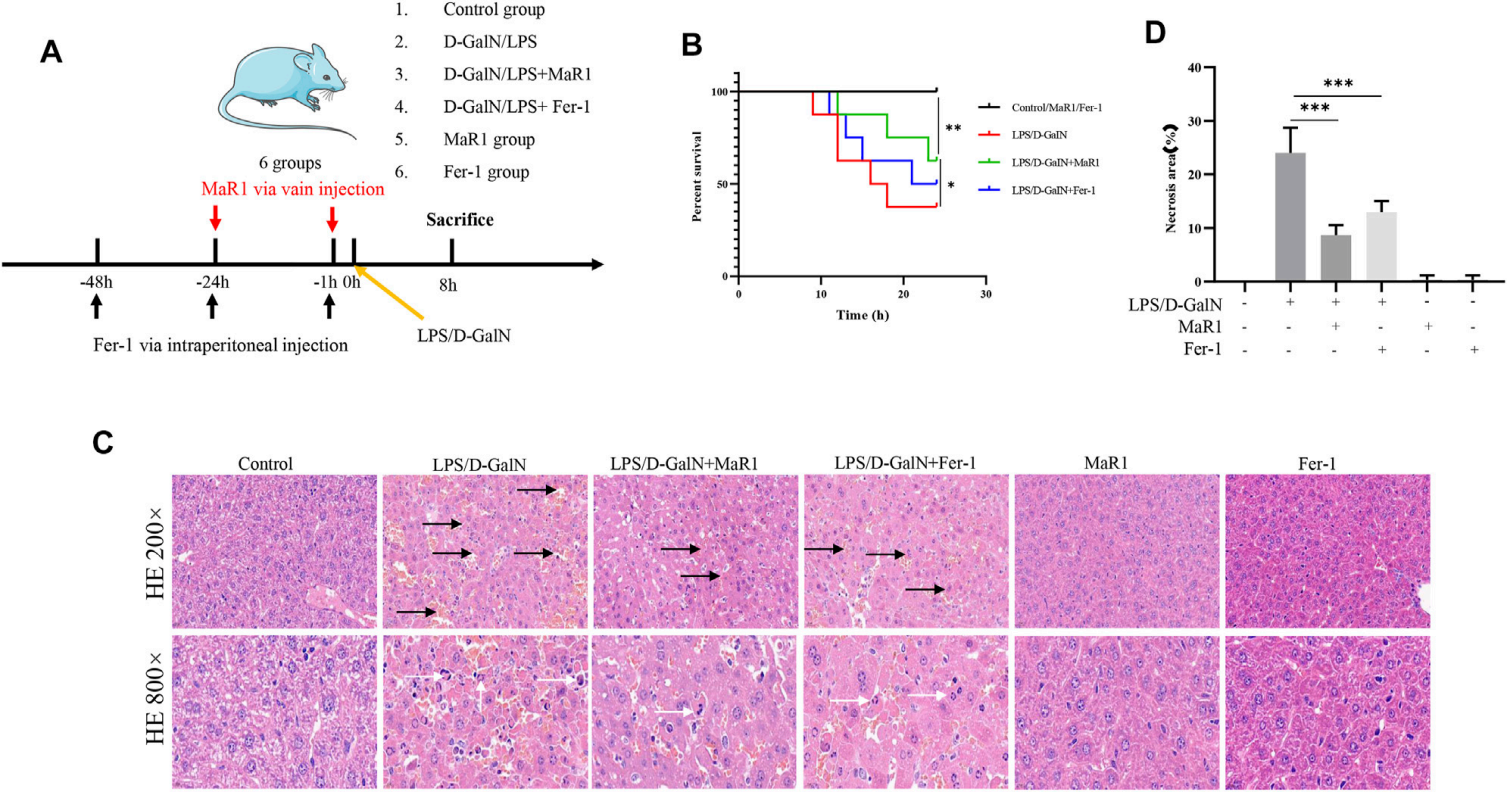
MaR1 and Fer-1 mitigated D-GalN/LPS-induced liver injury. **(A)** The treatments of mice. **(B)** The administration of MaR1 and Fer-1 significantly improved the median survival in mice treated with D-GalN/LPS. **(C,D)** The HE staining of liver tissue showed that D-GalN/LPS induced significant liver injury, while the administration of MaR1 and Fer-1 significantly mitigated D-GalN/LPS-induced liver injury. Black arrow showed necrotic tissue, and white arrow showed inflammatory cells. Data are shown as the mean ± SD, *p < 0.05; **p < 0.01; ***p < 0.001.

Male C57BL/6 mice aged 7–9 weeks, weighted 20–26 g were purchased from China Three Gorges University (Hubei, China). The mice were maintained on a 12 h light-dark cycle environment and are free access to water and food. Before experiments, the animals acclimated to the environment for a week. Mice were randomly divided into 6 groups (*n* = 8): 1) control group; 2) D-GalN (300 mg/kg, i.p.)/LPS (30 μg/kg, i.p.); 3) D-GalN/LPS + MaR1 (100ng, i.v.); 4) D-GalN/LPS + Ferrostatin-1 (Fer-1) (10 mg/kg, i.p.); 5) MaR1(100 ng); 6) Fer-1(10 mg/kg). The MaR1 and Fer-1 administrations were shown in Figure 1A. The dose and time of MaR1 administration were referenced to previous studies (Wang et al., 2015; Zhang et al., 2020). The survival status of mice within 24 h were observed. Then, the mice were divided into 6 groups again (*n* = 6), and the administration method was the same as before. The liver tissue and eye blood of the animals were taken at 8 h. The blood sample was centrifuged at high speed for subsequent experiments. One part of the liver tissue was fixed with formaldehyde, and the other part was frozen at −80°C. All animal study protocols were approved by the Institutional Animal Use and Care Committee of Huazhong University of Science and Technology. The number for ethics approval is S2661.

### Chemicals and Reagents

D-GalN/LPS which were used to create ALI models, were obtained from Sigma‐Aldrich (St. Louis, MO, United States). MaR1 was obtained from Cayman Chemical (Ann Arbor, MI, United States). Fer-1 and 3-(4,5)-dimethylthiahiazo (-z-y1)-3,5 diphenytetrazoliumromide (MTT) were obtained from Med Chem Express (New Jersey, United States). Erastin, a ferroptosis activator, was purchased from Selleck Chemicals (Houston, United States). The levels of serum alanine transaminase (ALT, C009-2-1), aspartate transaminase (AST, C010-2-1), reduced glutathione (GSH, A006-2-1), GSH//oxidized glutathione (GSSG), A061-1-2), malondialdehyde (MDA, A003-1-2), and serum iron assay (A039-1-1) were detected with kits (Nanjing Jiancheng Institute of Biotechnology, Nanjing, China). The level of serum IL-1β (1210122), TNF-α (1217202), and IL-6 (1210602) were detected with enzyme-linked immunosorbent assay (ELISA) (DAKEWE Bioengineering, Beijing, China). Antibodies against NF-κB p65 (8242), phosphorylated-p65 (3033), and Nrf2 (12721) were purchased from Cell Signaling Technology (Beverly, MA, United States). Antibodies against GAPDH (60004-1), and HO-1 (27282-1) were purchased from Proteintech (Wuhan, China). Antibody against GPX4 (ab125066) was obtained from Abcam (Shanghai, China).

### Cell Culture

HepG2 cells were obtained from Cell Bank of the Chinese Academy of Sciences (Shanghai, China). Cells were cultured in Dulbecco’s Modified Eagle’s Medium (Gibco) with 10% fetal bovine serum (FBS, Gibco) in a moist incubator with 5% CO_2_ at 37°C.

For studies investigating the inhibition of MaR1 in ferroptosis, MaR1 pretreated cells for 1 h, and then erastin or D-GalN were added for 6 h.

### Cell Viability Assay

MTT assay was used to determine cell viability. Briefly, HepG2 cells (1×10^3^ per well) in the logarithmic growth phase were plated into 96-well plates. Drugs were administered after 24 h. After different time processing, MTT reagent (10 μL/well) was added into wells. After 4 h, dimethyl sulfoxide (DMSO) was added and the plate was agitated for 20 min at 37°C. Finally, a microplate reader (Bio-Rad, Hercules, CA, United States) was used to measure the absorbance at 570 nm.

### Measurements of ALT and AST Levels

The levels of serum ALT and AST were measured under the guidance of the manufacturer’s instructions. Briefly, solubility curves were made based on standard samples. Next, the test solution, matrix solution, and chromogenic solution were added to the 96-well plate in different order. The 96-well plate was placed at room temperature for 15 min, the OD value of each well were measured with a microplate reader at 510 nm.

### Measurement of MDA, GSH, and GSH/GSSG Counts

The serum MDA, GSH and liver GSH/GSSG levels were measured using MDA, GSH and GSH/GSSG assay kit. Different reagents were added according to the operation, and the OD value of each well was measured with a microplate reader at 532 nm or 412 nm.

### Iron Assay

Firstly, serum and iron developer well were mixed as required. The mixture was boiled for 5 min at 100°C and then centrifuged at 3,500×*g* for 10 min to collect the supernatant. The supernatants were added into a 96-well plate and the absorbance was measured at 520 nm with a microplate reader.

### ROS Assay

ROS activities of cells were measured by a fluorescent probe (DCFH-DA) (Beyotime, Shanghai, China). HepG2 cells in a 6- well plate was treated with LPS or MaR1 for 6 h, then 2 μL/well DCFH-DA was added for 30 min at 37°C. Subsequently, the cells were washed three times with serum-free medium. The mean DCFH-DA was measured by flow cytometric analysis. The ROS activities of liver tissue were detected by dihydroethidium (DHE). The signals were quantified by fluorescence microscopy and ImageJ software (National Institutes of Health, Bethesda, MD).

### Histopathological Evaluation

Liver sections were stained with hematoxylin and eosin (H&E) for routine histology. Five random fields (200 × ) were selected in each slice, and the necrotic areas were evaluated by two pathologists and measured by ImageJ software.

### Oil Red O

Frozen liver sections were used for Oil Red O staining (Servicebio, Wuhan, China), and were performed according to the standard protocol. Staining intensity was quantified by fluorescence microscope and ImageJ software (Deutsch et al., 2014).

### dUTP Nick-End Labeling (TUNEL) Assay

The TUNEL assay was performed as described previously (Li et al., 2021). Five random fields (200 × ) were selected in each slice, and the positive cells were measured by ImageJ software.

### ELISA Assay

The serum levels of TNF-α, IL-1β, and IL-6 were determined using ELISA kits following the manufacturer’s instructions.

### Apoptosis Assay

The apoptosis was evaluated by apoptosis kit (BioLegend, California, United States). HepG2 cells were with LPS or MaR1 treatment in 6-well plates. After 24 h, the cells were washed for three time and the Annexin V-APC and 7-AAD antibodies were added before flow cytometry.

### Western Blot Analysis

Proteins were extracted from liver tissue or HepG2 cells. Nuclear protein extraction kit (Beyotime, P0027, Shanghai, China) was used to extract nuclear following the instruction. BCA protein assay (Beyotime, P0012, Shanghai, China) was used to measure protein concentration. Sodium dodecyl sulphate–polyacrylamide gel electrophoresis was used to separate proteins and the proteins were transferred to a polyvinylidene fluoride membrane (Millipore). 5% skim milk was used to block the membrane. The membrane was incubated with primary antibodies and secondary antibodies. The membrane was imaged with ECL reagents (Cell Signaling Technology, Beverly, MA, United States) by the imaging system (Thermo Fisher Scientific).

### Statistical Analysis

GraphPad Prism 8.0 (GraphPad Software, Inc., La Jolla, CA) was used for statistical analysis. Results were expressed as the mean ± standard deviation (SD) and the student’s *t*-test was applied to compare differences of two groups. For multiple comparisons, one-way analysis of variance (ANOVA) was used. Student’s *t*-test or one-way ANOVA followed by post-hoc test was used to determine statistically significant differences. *p* < 0.05 was considered statistically significant. *p < 0.05; **p < 0.01; ***p < 0.001.

## Results

### MaR1 and Fer-1 Mitigated D-GalN/LPS-Induced Liver Injury

The treatments of mice were shown in Figure 1A. As shown in Figure 1B, the administration of MaR1 and Fer-1 significantly improved the median survival in mice treated with D-GalN/LPS. The H&E staining of liver tissue showed that D-GalN/LPS induced significant liver injury, while the administration of MaR1 and Fer-1 significantly mitigated D-GalN/LPS-induced liver injury (Figures 1C,D).

### MaR1 Administration Protected Against Liver Injury and Inhibited Hepatocyte Death

**FIGURE 2:**
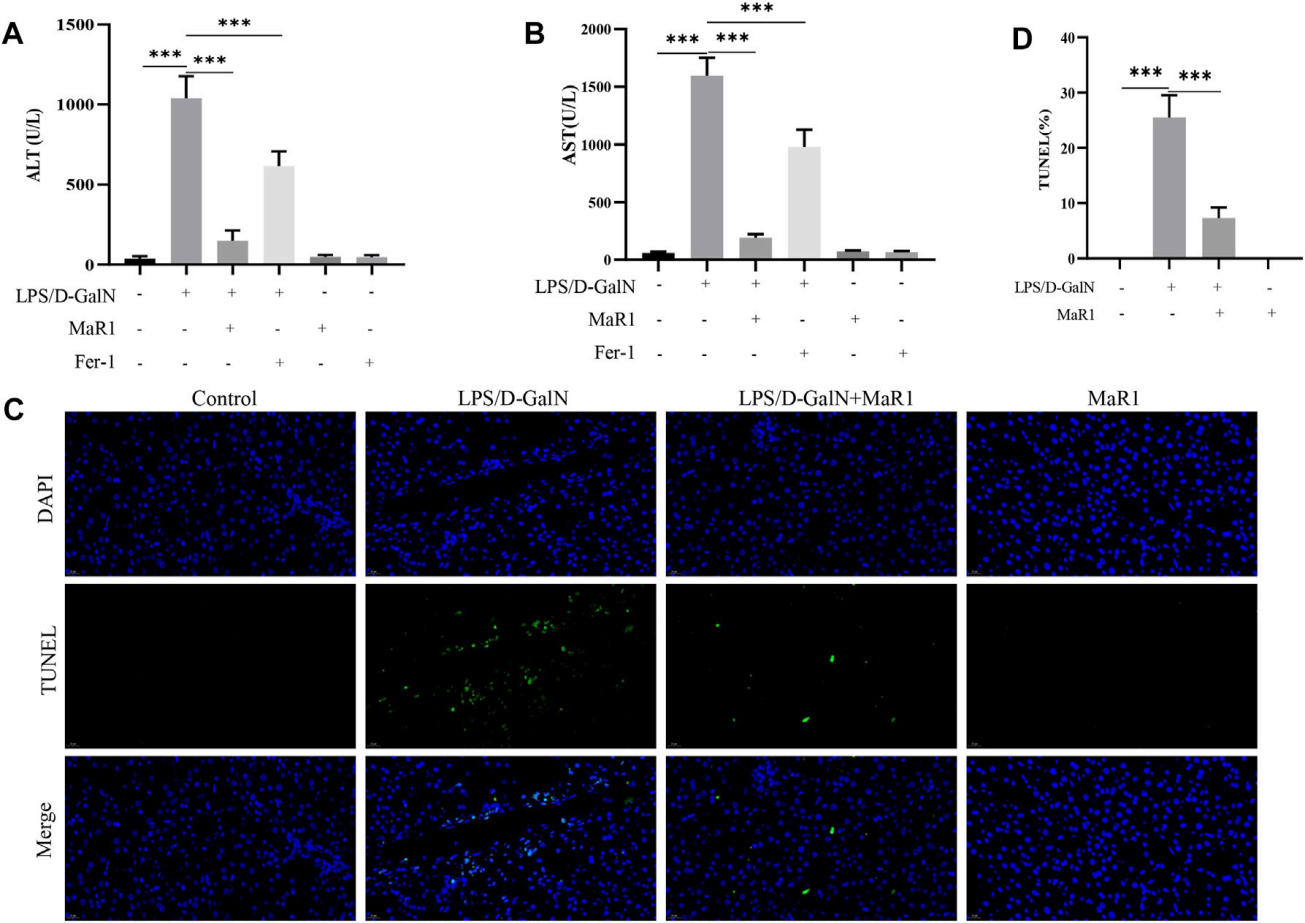
MaR1 administration protected against liver injury and inhibited hepatocyte death. **(A,B)** Serum ALT and AST levels. **(C,D)** TUNEL assay also showed that D-GalN/LPS promoted hepatocyte death, while MaR1 inhibited hepatocyte death caused by D-GalN/LPS. Data are shown as the mean ± SD, *p < 0.05; **p < 0.01; ***p < 0.001.

We measured serum ALT and AST levels. The results showed that D-GalN/LPS markedly increased the serum ALT and AST levels, while MaR1 administration dramatically reduced the levels of serum ALT and AST compared with D-GalN/LPS group (Figures 2A,B). TUNEL assay also showed that D-GalN/LPS promoted hepatocyte death, while MaR1 inhibited hepatocyte death caused by D-GalN/LPS (Figures 2C,D).

### MaR1 Inhibited D-GalN/LPS-Induced Liver Lipid Peroxidation in Mice

**FIGURE 3:**
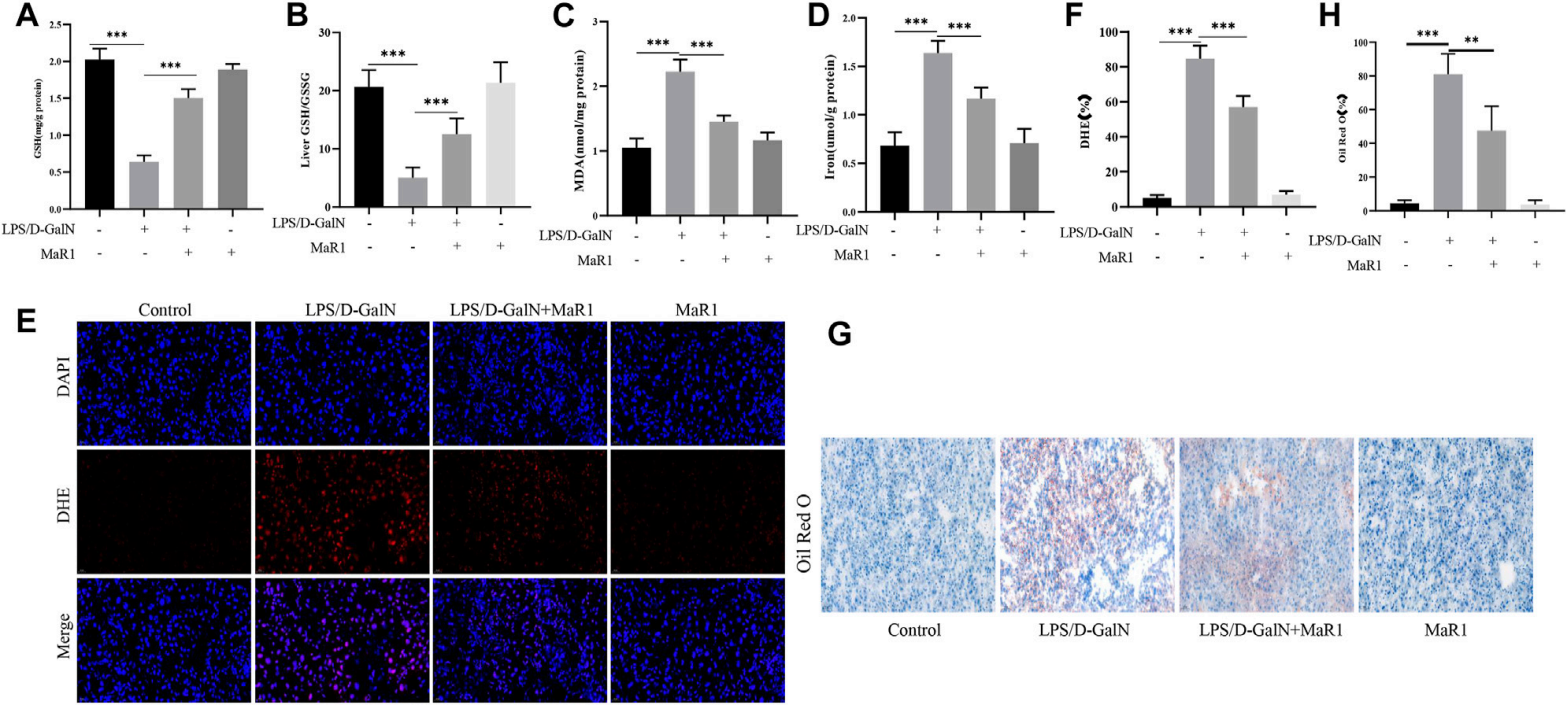
MaR1 inhibited D-GalN/LPS-induced liver lipid peroxidation in mice. **(A)** D-GalN/LPS significantly reduced the GSH content, and the treatment of MaR1 significantly increased the GSH content. **(B)** MaR1 significantly increased the GSH/GSSG in liver tissue. **(C)** D-GalN/LPS significantly increased the serum MDA content, while MaR1 significantly decreased the level of MDA. **(D)** Serum iron content significantly reduced after mice treated with MaR1. **(E,F)** MaR1 efficiently reversed the increase of ROS level. **(G,H)** Oil Red O staining showed that MaR1 significantly decreased the lipid accumulation in the liver with D-GalN/LPS treatment. Data are shown as the mean ± SD, *p < 0.05; **p < 0.01; ***p < 0.001.

GSH is an important antioxidant in the body, and the essence of ferroptosis is the depletion of GSH. We detected the GSH content in the serum, and the results showed that D-GalN/LPS significantly reduced the GSH content, and the treatment of MaR1 significantly increased the GSH content (Figure 3A). Then we tested the level of GSH/GSSG in liver tissue, and the results showed MaR1 significantly increased the level of GSH/GSSG in liver tissue (Figure 3B). MDA can reflect the degree of tissue lipid peroxidation damage. As shown in Figure 3C, D-GalN/LPS significantly reduced the serum MDA content. When the mice were treated with MaR1, the MDA level was significantly increased compared with D-GalN/LPS group. The iron content can indicate the degree of ferroptosis. The results showed the serum iron content of the mice in the D-GalN/LPS group was significantly increased compared with the control group, while the serum iron content significantly reduced after the mice treated with MaR1 (Figure 3D). ROS is an important activation signal for ferroptosis. We used DHE staining to detect the level of ROS in liver tissues. The results showed that compared with the control group, the ROS expression level of mice in the D-GalN/LPS group was significantly increased. And MaR1 efficiently reversed the increase of ROS level (Figures 3E,F). Oil Red O staining is used to detect fat content in tissue. The results of Oil Red O staining showed that D-GalN/LPS significantly increased the lipid accumulation, while MaR1 significantly decreased the lipid accumulation in the liver (Figures 3G,H).

### MaR1 Inhibited Ferroptosis-Induced Liver Injury Through Proinflammatory Factors Inhibition and Nrf2/HO-1/GPX4 Activation

**FIGURE 4:**
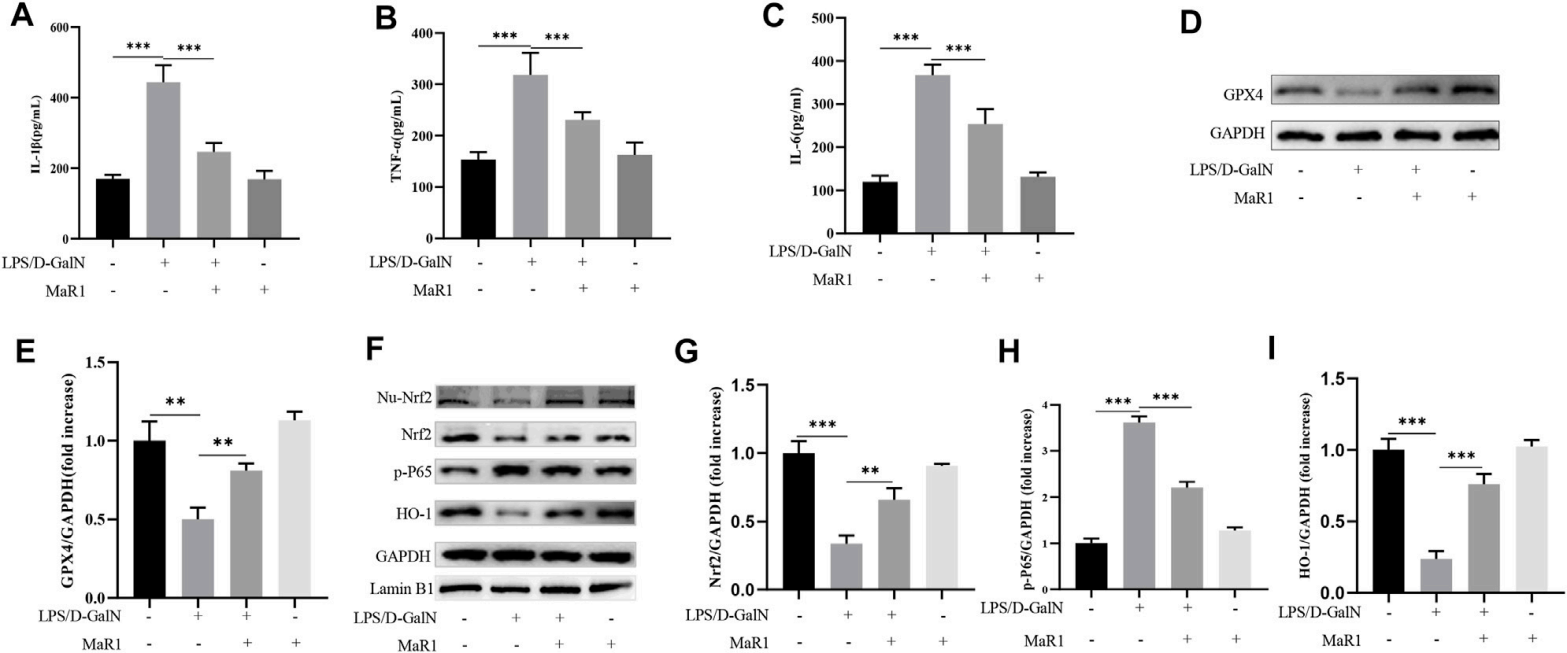
MaR1 inhibited ferroptosis-induced liver injury through proinflammatory factors inhibition and Nrf2/HO-1/GPX4 activation. **(A–C)** MaR1 significantly reduced the increase in serum IL-1β, TNF-α, and IL-6 levels caused by D-GalN/LPS. **(D,E)** Western blot showed that D-GalN/LPS significantly inhibited the expression of GPX4, and MaR1 attenuated this change. **(F–I)** Western blot showed MaR1 treatment significantly promoted the expression of Nrf2 and HO-1, and inhibited the expression of p-P65. Data are shown as the mean ± SD, *p < 0.05; **p < 0.01; ***p < 0.001.

ELISA kit was used to detect serum IL-1β, TNF-α, and IL-6 levels. The results showed that compared with the control group, D-GalN/LPS treatment significantly increased serum IL-1β, TNF-α, and IL-6 levels. MaR1 significantly reduced the increase in cytokine caused by D-GalN/LPS (Figures 4A–C). GPX4 is a key protein to inhibit ferroptosis, and the decrease of GPX4 activity promotes ferroptosis. Subsequently, we extracted liver tissue proteins, western blot showed that D-GalN/LPS significantly inhibited the expression of GPX4, and MaR1 attenuated this change (Figures 4D,E). At the same time, western blot showed that D-GalN/LPS promoted the expression of p-P65 and inhibited the expression of Nrf2 and the downstream molecule HO-1. MaR1 treatment significantly promoted the expression of Nrf2 and HO-1, and inhibited the expression of p-P65 **(** Figures 4F–I).

### MaR1 Suppressed LPS-Induced and Erastin-Induced Ferroptosis *In vitro*

**FIGURE 5:**
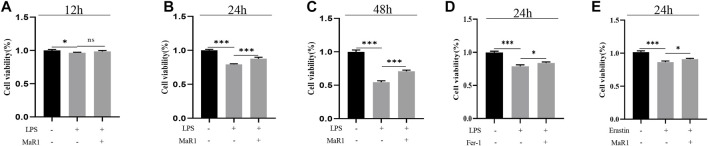
MaR1 suppressed LPS-induced and erastin-induced ferroptosis *in vitro*. **(A–C)** MTT assay showed that MaR1 inhibited HepG2 cell death caused by LPS at 12, 24, and 48 h. **(D)** Fer-1 inhibited LPS-induced cell death. **(E)** MaR1 inhibited cell death caused by erastin. Data are shown as the mean ± SD, *p < 0.05; **p < 0.01; ***p < 0.001.

We further explore the understanding mechanism of MaR1 in inhibiting ferroptosis *in vitro*. MTT assay showed that MaR1 (10 nM) inhibited HepG2 cell death caused by LPS (1 μg/ml) at 12, 24, and 48 h (Figures 5A–C). To further verify that LPS cause ferroptosis, we treated cells with LPS and Fer-1, the results showed that Fer-1 inhibited LPS-induced cell death, indicating LPS caused ferroptosis (Figure 5D). Finally, we treated cells with erastin, a ferroptosis activator, and the results showed that MaR1 inhibited cell death caused by erastin (10 μM), indicating MaR1 inhibited ferroptosis (Figure 5E).

### MaR1 Suppressed D-GalN-Induced and Erastin-Induced Ferroptosis *In vitro*

**FIGURE 6:**
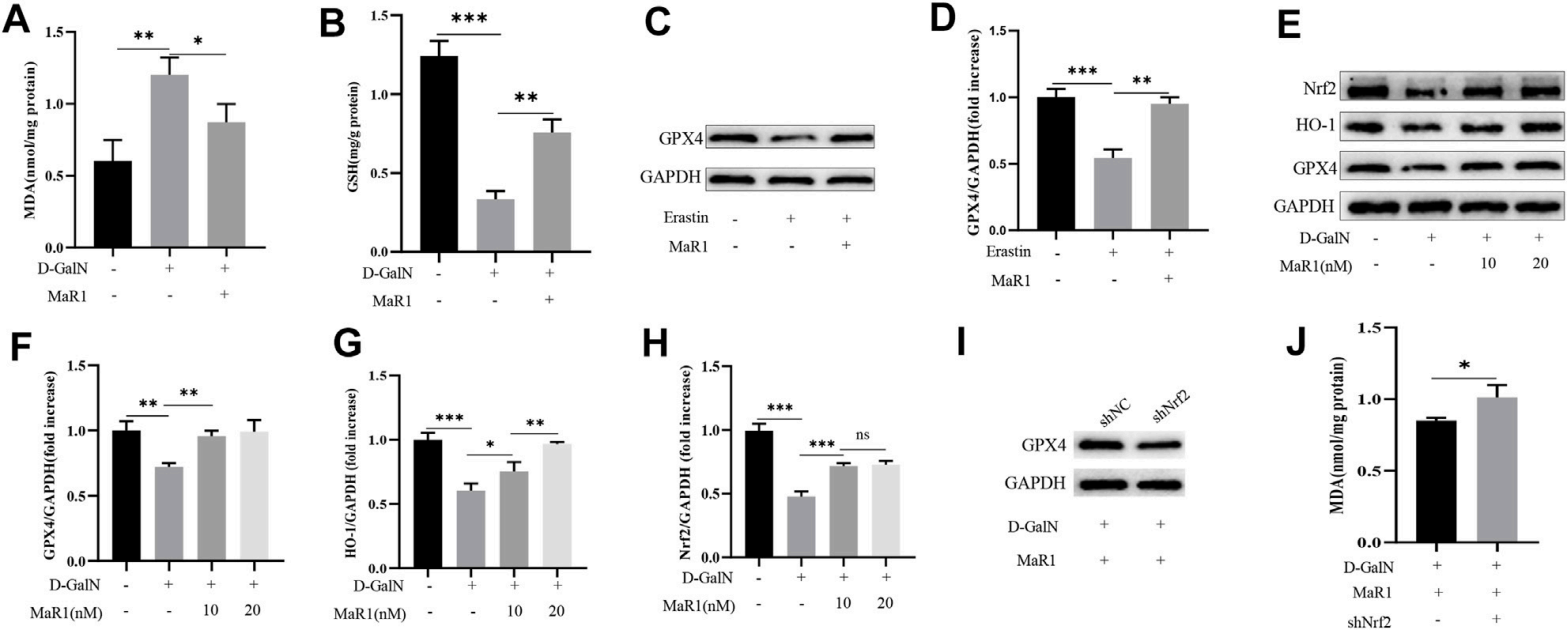
MaR1 suppressed D-GalN-induced and erastin-induced ferroptosis *in vitro*. **(A)** D-GalN significantly increased the MDA content, while MaR1 administration reduced the MDA content. **(B)** The test of GSH content in the cells. **(C,D)** Erastin significantly reduced the expression of GPX4, while MaR1 promoted the expression of GPX4. **(E–H)** MaR1 promoted the expression of Nrf2, HO-1, and GPX4. **(I)** When Nrf2 was knocked down, the expression of GPX4 significantly decreased. **(J)** The level of MDA significantly increased in knock-down group. Data are shown as the mean ± SD, *p < 0.05; **p < 0.01; ***p < 0.001.

We treated HepG2 cells with D-GalN (50 mM) or MaR1 for 6 h and then detected the cell MDA content. The results showed that D-GalN significantly increased the MDA content, while MaR1 administration reduced the MDA content (Figure 6A). Next, we tested the GSH content in the cells, and the results also showed that D-GalN increased the GSH content, while MaR1 significantly reduced the GSH content (Figure 6B). Subsequently, we treated the cells with erastin or MaR1 for 6 h to extract cell protein. Western blot results showed that erastin significantly reduced the expression of GPX4, while MaR1 promoted the expression of GPX4 (Figures 6C,D). At the same time, western blot showed that D-GalN significantly reduced the expression of Nrf2, HO-1, and GPX4, while MaR1 promoted the expression of these proteins (Figures 6E–H). To further illustrate that MaR1 acts on Nrf2, we used lentivirus to knock down *Nrf2.* The western blot showed the expression of GPX4 significantly decreased in knock down group, while the level of MDA significantly increased (Figures 6I,J).

### MaR1 Inhibited Ferroptosis by Inhibiting ROS Production in HepG2 Cells

**FIGURE 7:**
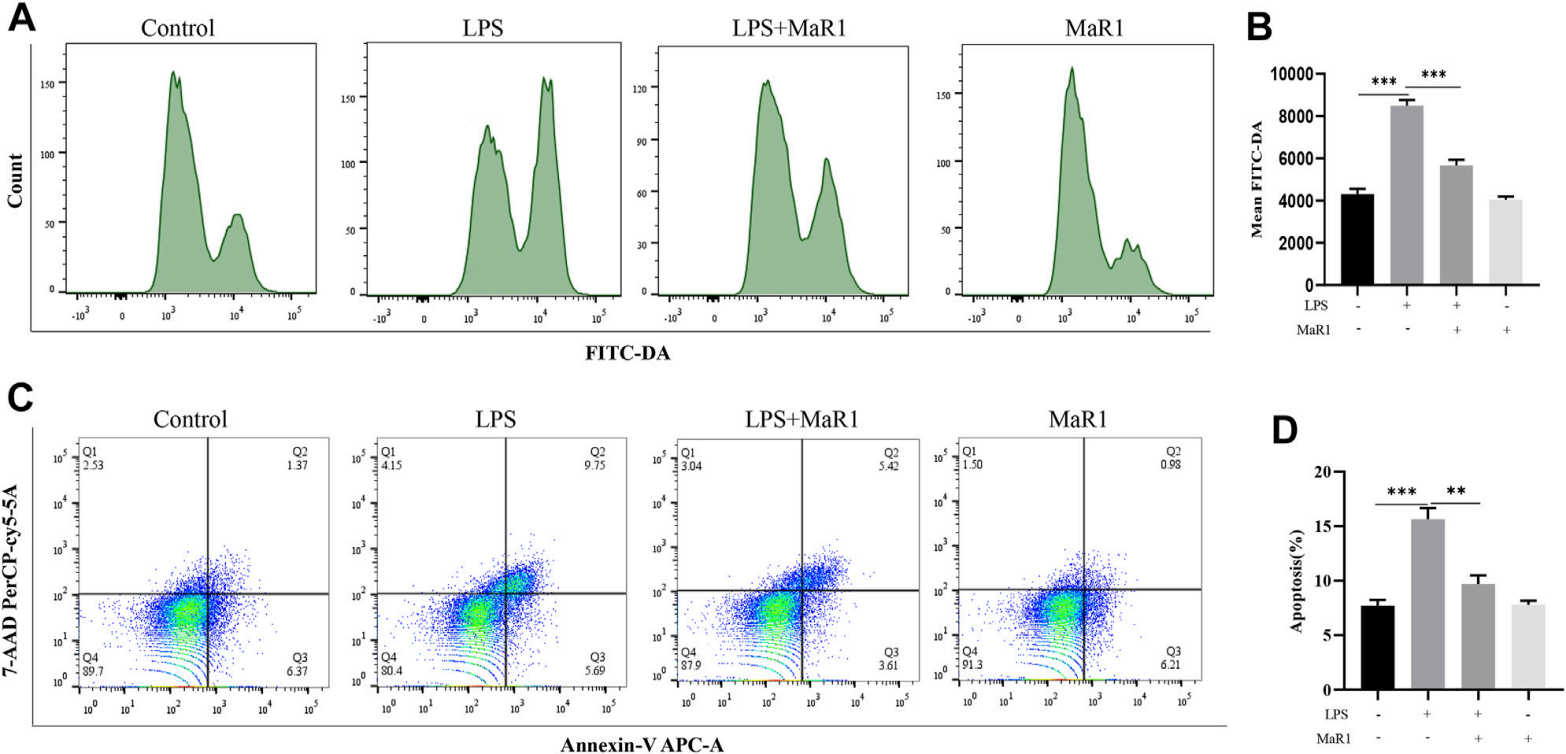
MaR1 inhibited ferroptosis by inhibiting ROS production *in vitro*. **(A,B)** The flow cytometry showed that LPS significantly promoted the production of ROS, while MaR1 significantly reduced the ROS production. **(C,D)** MaR1 inhibited LPS-induced apoptosis at 24 h. Data are shown as the mean ± SD, *p < 0.05; **p < 0.01; ***p < 0.001.

As shown in the previous results, MaR1 inhibited ferroptosis by activating Nrf2 and GPX4, and ROS is an important activator of ferroptosis. Therefore, we hypothesized that MaR1 inhibited the production of ROS by activating Nrf2 and GPX4, thereby inhibiting ferroptosis. We used flow cytometry to detect the expression level of ROS, and the results showed that LPS significantly promoted the production of ROS, while MaR1 significantly reduced the ROS production, which validated our hypothesis (Figures 7A,B). In addition, flow cytometry showed that MaR1 inhibited LPS-induced apoptosis at 24 h (Figures 7C,D).

## Discussion

LPS and D-GalN are recognized as harmful substances that cause ALI, while the current treatments are not effective in treating ALI. Therefore, it is essential to further explore the mechanism of D-GalN/LPS -induced hepatotoxicity and develop potential drugs for the treatment of ALI. In the present study, we reported another innovative cell death mode-ferroptosis also contributed to ALI. And we found that MaR1 effectively attenuated both D-GalN/LPS-induced and erastin-induced liver injury by inhibiting ROS and activating Nrf2/HO-1/GPX4 pathways.

Cell death is closely related to the growth, development, and senescence of organisms. The way of programmed cell death usually includes apoptosis, necroptosis, pyroptosis, and so on (Osbron and Goodman, 2022). Stockwell et al. found that erastin, a new compound that could induced cell death in a different way from the known cell death methods, and proposed a concept of iron-dependent programmed cell death (Dixon et al., 2012). The mechanism of ferroptosis is that the membrane phospholipids form lipid ROS under non-enzymatic or lipoxygenase pathways (Park and Chung, 2019; Chen et al., 2021a). In this study, we used D-GalN/LPS to create a liver injury model and found that ferroptosis inhibitor (Fer-1) alleviated liver injury, which suggested that ferroptosis occurred in the liver injury model induced by D-GalN/LPS. In addition, we found the level of IL-1β and TNF-α significantly increased in D-GalN/LPS group. The source of serum cytokines may be hepatocyte cells, macrophage cells, or fibroblast cells. Macrophages play a key role in inflammatory response and are the main effector cells, which can release a large number of inflammatory mediators in the liver injury (Feng et al., 2018; Giavridis et al., 2018). Surely, hepatocyte cells and fibroblast cells can also produce cytokines under the external stimulus. In the D-GalN/LPS-induced liver injury model, LPS is more likely to act on macrophages and cause the release of inflammatory factors. D-GalN is more likely to act on hepatocyte cells and lead to biosynthesis disorder. The pro-inflammatory factors caused by macrophages can further act on hepatocytes and promote ROS production in hepatocytes, or further activate macrophages, and aggravate the occurrence of ferroptosis.

The sensitivity of cells to ferroptosis is mainly regulated by ROS and GPX4 (Friedmann Angeli et al., 2014). GPX4 can convert the toxic lipid ROS to nontoxic lipid alcohol in the presence of GSH, thereby preventing ferroptosis (Shin et al., 2018; Seibt et al., 2019). Regarding the source of ROS, both hepatocyte cells and macrophage cells can produce ROS. D-GalN act on hepatocyte cells and inhibit mitochondrial biosynthesis, leading to the ROS production. Meanwhile, LPS can also increase ROS production by acting on macrophage cells, which mainly causes the release of inflammatory factors. Together they promote ferroptosis in hepatocytes. DHE staining showed that D-GalN/LPS significantly increased the level of ROS. Then, western blot showed that D-GalN/LPS obviously reduced the expression of GPX4 and promoted ferroptosis. GSH is an important antioxidant in the body. It not only converts H_2_O_2_ into H_2_O, scavenges free radicals, and maintains the balance of intracellular free radical content, but also acts as the cofactor of GPX4 participates in the reduction reaction. Studies have showed that GSH act against Kupffer cell-generated ROS, thereby protecting the hepatic infrastructure (Fang et al., 2020a; Wang et al., 2020b). Under adverse stimuli, GSH can be converted into GSSG, and the ratio of GSH/GSSG is an excellent biomarker for redox homeostasis in liver. The depletion of GSH will cause the GPX4 inactivation, increase the lipid peroxidation in the cell, and lead to ferroptosis (Proneth and Conrad, 2019; Chen et al., 2021b). We tested the serum GSH content and found that D-GalN/LPS significantly reduced GSH expression, which was consistent with some research reports (Wang et al., 2018; Ji et al., 2021). Therefore, D-GalN/LPS may cause GPX4 inactivation by causing GSH deficiency. Free Fe^2+^ is extremely oxidative, and it is easy to cause Fenton reaction with H_2_O_2_, which can cause oxidative damage to DNA, protein, and membrane lipids, and promote the occurrence of lipid peroxidation (Li et al., 2020b; Yang et al., 2021). In this study, we found that the serum iron content of the D-GalN/LPS group significantly increased. These results further indicated that ferroptosis occurred in D-GalN/LPS-induced ALI model.

MaR1, a new family of special pro-resolving mediators, has the ability to regulate inflammatory disorders and possess pro-resolution properties. The effects of MaR1 were linked with the ability to inhibit neutrophil recruitment and reduce ROS and proinflammatory cytokines production. Numerous studies have shown that MaR1 exerts potent protective effects in many diseases (Tang et al., 2017; Jin et al., 2018; Huang et al., 2020). In the present study, we found that MaR1 significantly increased the survival rate of mice with ALI induced by D-GalN/LPS, reduced serum ALT, AST, IL-1β, and TNF-α levels, and protected against liver injury. At the same time, we found that MaR1 significantly increased serum GSH, and reduced MDA and iron content. Western blot also showed that MaR1 increased the expression of GPX4, thereby inhibiting ferroptosis. *In vitro* experiments, we found MaR1 increased the expression of GPX4 and inhibited D-GalN/LPS-induced and erastin-induced ferroptosis. The increase of intracellular ROS level is an important inducing factor for ferroptosis, which is also the reason that lipid antioxidants can inhibit ferroptosis. Mitochondria, as iron-rich organelles mainly producing ROS, are considered important places for ferroptosis (Wang et al., 2020c). We used DHE staining to mark the expression level of ROS in liver tissues, and the results showed that MaR1 significantly reduced the level of ROS in tissues. TUNEL staining also showed that MaR1 reduced liver cell death caused by D-GalN/LPS. *In vitro* experiments, the results showed that MaR1 obviously reduce the expression of ROS caused by LPS, promoted GPX4 expression. Therefore, the mechanism of MaR1 protecting the liver may be inhibiting the ROS and pro-inflammatory factors production, and activating GPX4.

Nrf2, a crucial transcription factor, regulates the oxidative stress of cells, and it is also an important regulator of maintaining intracellular redox homeostasis (Pillai et al., 2022; Ulasov et al., 2022). Nrf2 can induce and regulate the constitutive and inducible expression of antioxidant proteins, and reduce cell damage caused by ROS, and maintain the body’s redox homeostasis (Zhuang et al., 2019; Tao et al., 2022). Under normal physiological conditions, Nrf2 exists in the cytoplasm, it connects with Keap1 and maintains Nrf2 at a low level. When receiving external stimuli, Nrf2 dissociates from Keap1, and after Nrf2 transfers to the nucleus, it binds to the promoter region, activates the downstream molecule HO-1, and exerts an anti-inflammatory effect. Studies have shown that Nrf2 activation inhibited ROS production (Zhang et al., 2019; Fang et al., 2020b). Therefore, we further explored whether MaR1 inhibited ROS production by activating Nrf2/HO-1, thereby inhibiting ferroptosis. The results of tissue protein showed that MaR1 promoted the expression of Nrf2 and HO-1. *In vitro* experiments, we also found that MaR1 promoted the expression of Nrf2 and HO-1. When *Nrf2* was knocked down, the protective effect of MaR1 significantly decreased. Therefore, we hypothesized that MaR1 inhibited ROS production by activating Nrf2/HO-1, thereby inhibiting ferroptosis. Our study was the first to report that MaR1 protected against liver injury by inhibiting ferroptosis.

Surely, this study had several limitations. First, we only explored the effect of MaR1 on Nrf2/HO-1/GPX4 signaling pathway, while there are other pathways affecting ferroptosis. Second, we used hepatocellular carcinoma cells instead of normal hepatocyte cells for the vitro experiments. Finally, the proportion of MaR1 inhibiting liver injury by inhibiting ferroptosis remains to be further explored.

In conclusion, our current study provided a new perspective that ferroptosis was involved in the development and progression of D-GalN/LPS-induced ALI. More importantly, we reported that MaR1 protected against ferroptosis-induced liver injury through ROS inhibition and Nrf2/HO-1/GPX4 activation. Therefore, MaR1 can be considered a promising drug for the therapy of D-GalN/LPS-induced ALI.

## Data Availability

The original contributions presented in the study are included in the article/Supplementary Materials, further inquiries can be directed to the corresponding authors.

## Abbreviations

ALI, acute liver injury; ALT, alanine transaminase; AST, aspartate transaminase; DMSO, dimethyl sulfoxide; D-GalN, D-galactosamine; ELISA, enzyme-linked immunosorbent assay; Fer-1, Ferrostatin-1; GSH, reduced glutathione; GPX4, glutathione peroxidase 4; H&E, hematoxylin and eosin; LPS, lipopolysaccharide; MaR1, Maresin1; MDA, malondialdehyde; MTT, 3-(4,5)-dimethylthiahiazo (-z-y1)-3,5 diphenytetrazoliumromide; NF-κB, nuclear factor-kappa B; ROS, reactive oxygen species; SD, standard deviation; TUNEL, dUTP nick-end labeling.
